# Relationship between myocardial infarction and atrial fibrillation: A bidirectional Mendelian randomization study

**DOI:** 10.1097/MD.0000000000040252

**Published:** 2024-11-01

**Authors:** Jin Rao, Yue Yu, Pengchao Cheng, Xuefu Wang, Pei Wang, Zhinong Wang

**Affiliations:** a Department of Cardiothoracic Surgery, Changzheng Hospital, Naval Medical University, Shanghai, China; b School of Health Science and Engineering, University of Shanghai for Science and Technology, Shanghai, China.

**Keywords:** atrial fibrillation, causal association, genome-wide analysis study, inverse-variance weighted, Mendelian randomization, myocardial infarction

## Abstract

Many studies have shown that myocardial infarction (MI) is significantly associated with atrial fibrillation (AF), but the causal relationship between MI and AF has not been established. Therefore, we performed this Mendelian randomization (MR) study to investigate the relationship between MI and AF. We used a publicly available summary statistical dataset for MI based on genome-wide analysis studies (GWAS; ebi-a-GCST011364; 14,825 cases and 2680 controls) and a summary statistical dataset for AF based on an European GWAS (finn-b-I9_AF_REIMB; 10,516 cases and 116,926 controls). The 2‐sample bidirectional MR analysis was performed using the inverse-variance weighted (IVW), MR-Egger, and weighted median methods. The causal effect of MI on AF was analyzed using 30 MI-specific single nucleotide polymorphisms (SNPs) that were characterized as instrumental variables (IVs) based on the GWAS data. The causal effect of MI on AF was confirmed by the IVW (odds ratio [OR] 1.42; 95% confidence interval [CI] 1.27–1.58; *P* < .001), MR-Egger (OR: 1.49; 95% CI: 1.15–1.93; *P* = .005), and weighted median (OR: 1.42; 95% CI: 1.24–1.63; *P* < .001) analyses. Furthermore, in the reverse MR analyses, the causal effect of AF on MI was analyzed using 20 AF-specific SNPs that were screened as IVs. The causal effect of AF on MI was significant based on the results from the IVW method (OR: 1.05; 95% CI: 1.00–1.09; *P* = .033). In conclusion, the bidirectional MR analyses demonstrated a clear bidirectional causal association between MI and AF.

## 1. Introduction

Myocardial infarction (MI) and atrial fibrillation (AF) are 2 of the most common cardiovascular diseases worldwide.^[1]^ MI is caused by the obstruction of coronary arteries, which interrupts blood flow in the affected area resulting in the death of the cardiac muscle cells; AF is defined as an arrhythmia caused by irregular and rapid heartbeats.^[2,3]^ Both MI and AF are associated with significant morbidity and mortality. MI is the leading cause of death worldwide, and AF is associated with an increased risk of stroke and heart failure.^[4,5]^ Furthermore, both MI and AF are associated with several common risk factors, including hypertension, diabetes, smoking, and obesity.^[6,7]^ However, the causal relationship between the 2 diseases is still unclear because of the complexity and heterogeneity of these 2 cardiovascular diseases.^[8,9]^

Mendelian randomization (MR) method utilizes genetic variants as instrumental variables (IVs) to infer the causal relationships between exposures and outcomes.^[10]^ This method has become increasingly popular in epidemiological studies because it provides more reliable causal inferences than the conventional observational studies.^[11]^ The results of MR studies may be clinically important for the prevention and treatment of human diseases because they establish the causal relationships between modifiable risk factors and disease outcomes.^[12]^ In recent years, several MR studies have shown associations between cardiovascular diseases and genetic variants as well as modifiable risk factors, including correlations between smoking and MI, and hypertension and AF.^[13,14]^ These findings suggest the existence of causal relationships between multiple risk factors and cardiovascular diseases. Previous MR studies have mostly focused on identifying the unidirectional causal relationships between risk factors and human diseases, including cardiovascular diseases.^[15,16]^ However, the bidirectional causal relationship between MI and AF has not been thoroughly investigated.

Therefore, in the present study, we aim to investigate the possible causal relationships between MI and AF by MR methods using genetic variants associated with MI or AF as IVs. The research findings might be beneficial in developing more effective strategies for the prevention and treatment of these diseases in the future.

## 2. Materials and methods

### 2.1. Overall study design

The data for this study was obtained from previously published GWAS that were approved by the corresponding institutional review boards; informed consent was obtained from all the participants in the original studies.^[17]^ Therefore, no further approvals were required in this study.^[18]^ The cause-and-effect relationships between MI and AF were analyzed using the 2-sample MR study, and single-nucleotide polymorphisms (SNPs) were defined as the IVs. SNPs can be used to model randomized controlled trials (RCTs) to determine the causal relationship between exposures and outcomes.

### 2.2. Genetic instrument variants for the exposure analysis

MI data was obtained from the GWAS by Hartiala et al,^[19]^ that included 14,825 participants who had experienced MI and 2680 control participants. SNPs were selected using the following criteria: a strong link with MI based on a genome-wide significance of *P* < 5 × 10^−8^; independence from other SNPs and lack of bias due to linkage disequilibrium (LD) was ensured by selecting the SNPs related to MI with an *r*² < 0.001 and a window size of 10,000 kb; and the correlation between IVs and exposure factors was estimated using the *F* statistic, which was calculated using the following formula: *F* statistic = (β/SE).^[2]^ In general, IVs with an *F* statistic value of >10 were regarded as unbiased.

### 2.3. Atrial fibrillation dataset for outcome analysis

The data regarding AF was obtained from the FinnGen project (FinnGen) that included 10,516 cases and 116,926 controls of European ancestry (https://gwas.mrcieu.ac.uk/datasets/finn-b-I9_AF_REIMB/).

### 2.4. Statistical analysis

MR analysis^[20]^ was performed with the following 3 assumptions: IVs were closely related to MI; AF was only affected by IVs associated with MI; and absence of confounders in the relationship between MI and AF that was based on the analyses of IVs. However, it was plausible that the results could be affected by genetic variations through a single pathway rather than being affected by other exposures (horizontal pleiotropy), which do not conform to the assumptions of MR and may cause bias to the causal estimates. Three different analytical methods based on the horizontal multiplicity model were used in the MR analysis to prevent bias due to horizontal pleiotropy. The results were considered as credible if consistent results were obtained using these 3 methods. The main analysis was performed using the inverse variance weighting (IVW) approach, which provided the most accurate estimates by assuming that all the SNPs were valid IVs.^[21]^ If one or more SNPs did not conform to the assumptions for the IVs, the random effects IVW approach was used because it weighed each rate according to the standard error and considered the possibility of heterogeneity. The weighted median method required at least 50% of the SNPs or IVs to be valid for providing a consistent estimate.^[22]^ After sorting the included SNPs based on their weights, the median of the corresponding distribution function was estimated according to the results of the experiments. Furthermore, if the genetic instrument was independent of the pleiotropic effects, MR-Egger regression method was used to determine an effect estimate.^[23]^ In this scenario, the pleiotropic effect was assessed according to the value of the MR-Egger’s intercept. If the MR-Egger’s intercept was closer to zero, pleiotropic effects or directional multiplicative effects were considered to be absent.^[24]^ Data processing and analysis were performed using R (4.3.2), along with Zstats v1.0 (www.zstats.net).

### 2.5. Sensitivity analysis

**Figure 1.**
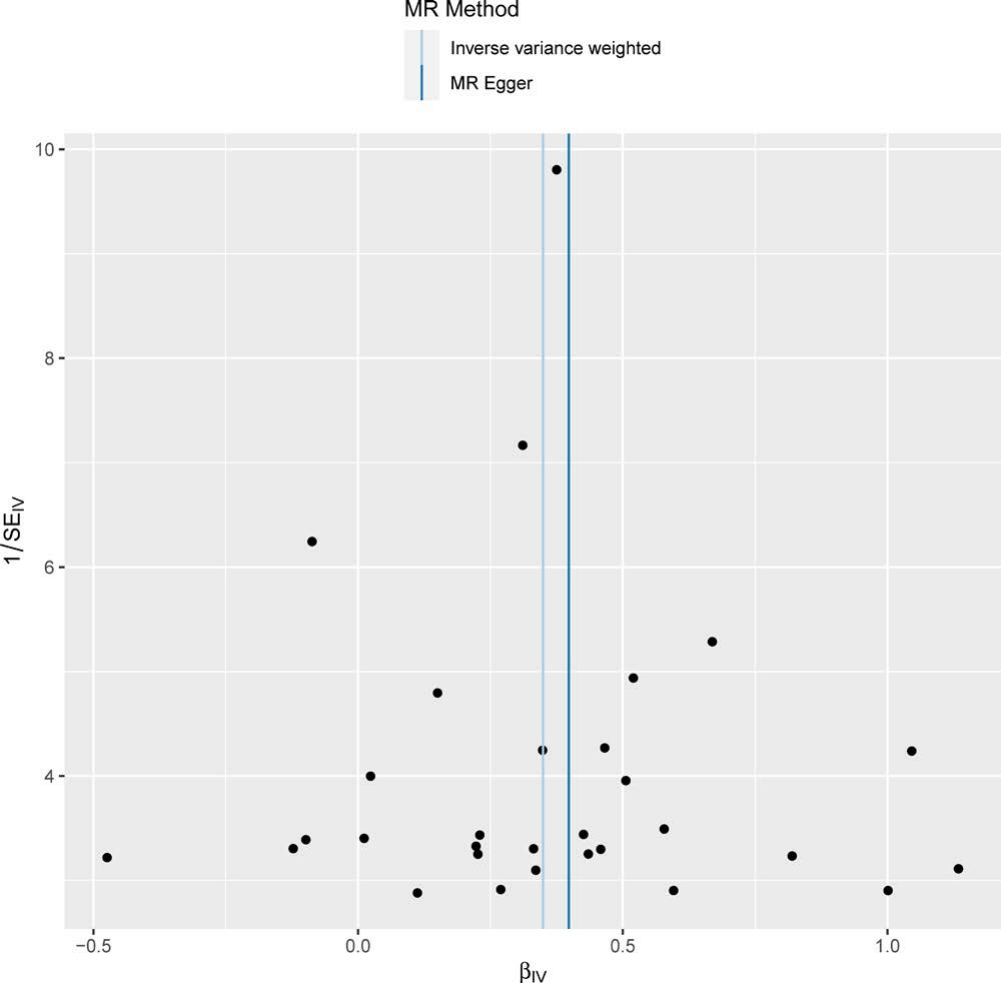
Funnel plot shows the overall heterogeneity of the Mendelian randomization (MR) estimates for the IVs. The light blue line represents the IVW estimate, and the dark blue line represents the MR-Egger estimate. IVW = inverse-variance weighted, MR = Mendelian randomization.

**Figure 2.**
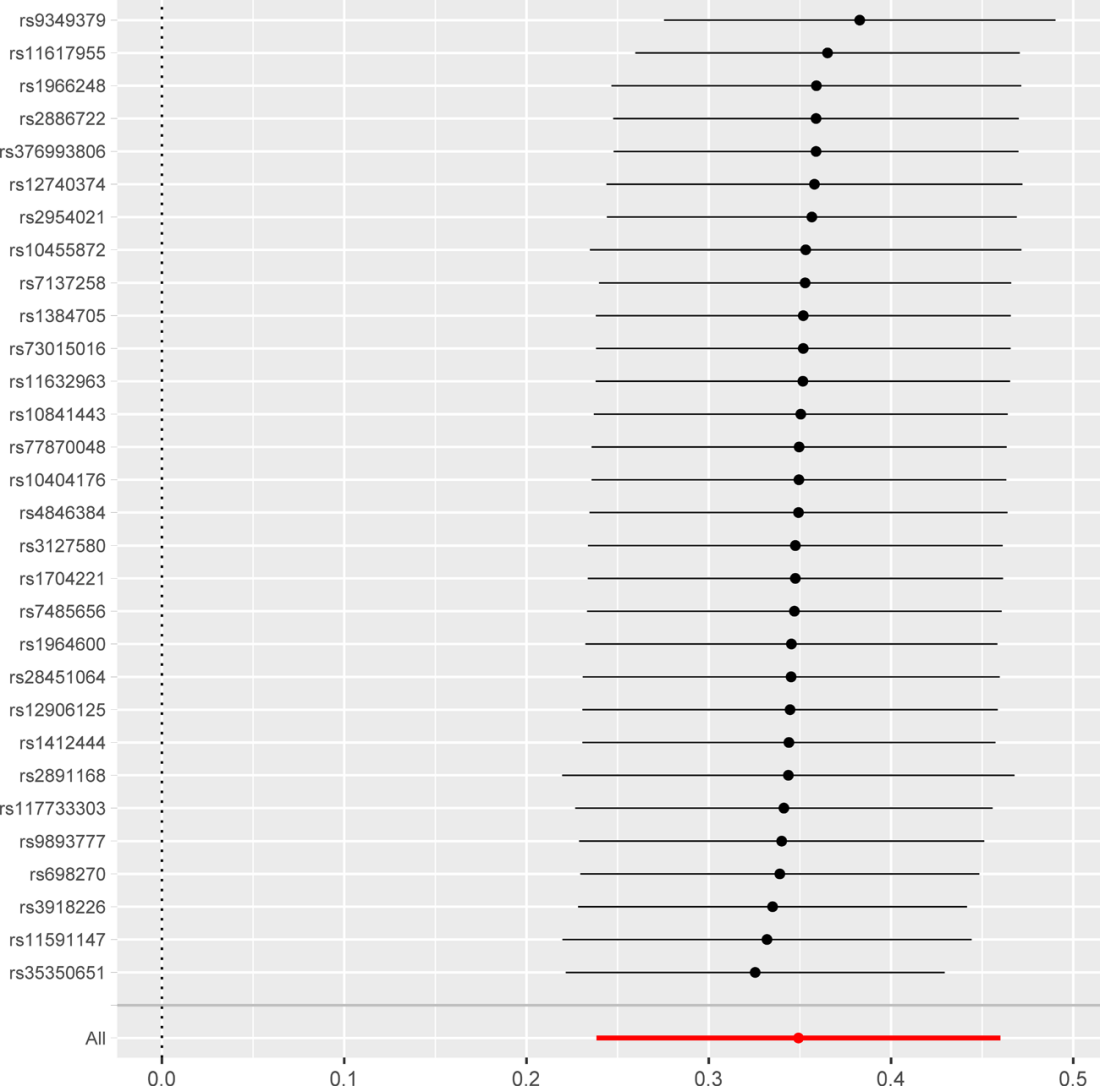
Sensitivity analysis shows the causal relationship between MI and AF based on unique single nucleotide polymorphisms associated with AF. AF = atrial fibrillation.

Funnel plots were used to display the individual Wald ratios of each SNP to determine the directional level pleiotropy of the IVs based on the degree of asymmetry or symmetry of the funnel plot. However, detection of horizontal pleiotropy for a small number of IVs included in the MR analyses was difficult using the funnel plots. The causal effect was evaluated based on the symmetry of the funnel plot (Fig. 1). Leave-one-out analysis was performed to investigate whether the estimates obtained from the IVW analyses were biased or dictated by individual SNPs during the meta-analyses. The leave-one-out analysis involved repeating the MR-IVW analyses for the SNPs after omitting a single SNP each time. The causal relationship for all the SNPs was considered significant if the results remained consistent (Fig. 2). The second assumption of the MR analysis was that the effects of the SNPs occurred only through modification of the exposure of interest in the absence of confounding factors. MR-Egger regression analysis was performed to estimate the horizontal pleiotropy based on the intercept and the *P* value. The absence of horizontal pleiotropy was ascertained if the intercept of the MR-Egger regression curve was close to zero and *P* > .05. This further indicated that pleiotropy did not bias the causal effect. Furthermore, in the published GWAS, there was no evidence that the included MI-associated SNPs were significantly associated with any phenotype except MI. This implied that the third assumption of the MR was valid. Consequently, there was no evidence that the genetic instruments of the 30 MI-associated SNPs were significantly associated with any other phenotype on a genome-wide scale. This was also consistent with the validity of the third MR assumption. The “Two sample MR” (version 0.5.6) software package was used for the MR analyses and the sensitivity analysis was performed using the R statistical software, version 3.6.2 (R Foundation for Statistical Computing, Vienna, Austria).^[25]^

## 3. Results

### 3.1. Characterization of instrumental variables for myocardial infarction

**Figure 3.**
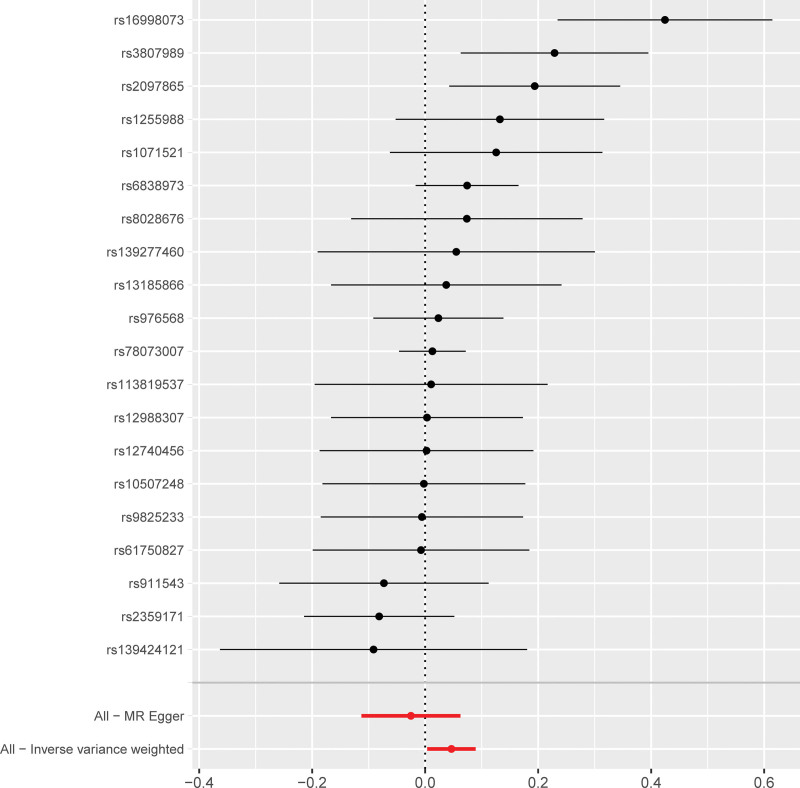
Forest plot shows the causal effects of MI-associated SNPs on AF. The black lines represent the effect sizes of all the MI-related SNPs. The combined significance of the IVs based on the MR-Egger test and the IVW method are shown as red lines at the bottom. AF = atrial fibrillation, IVW = inverse-variance weighted, MI = myocardial infarction, MR = Mendelian randomization, SNPs = single nucleotide polymorphisms.

**Figure 4.**
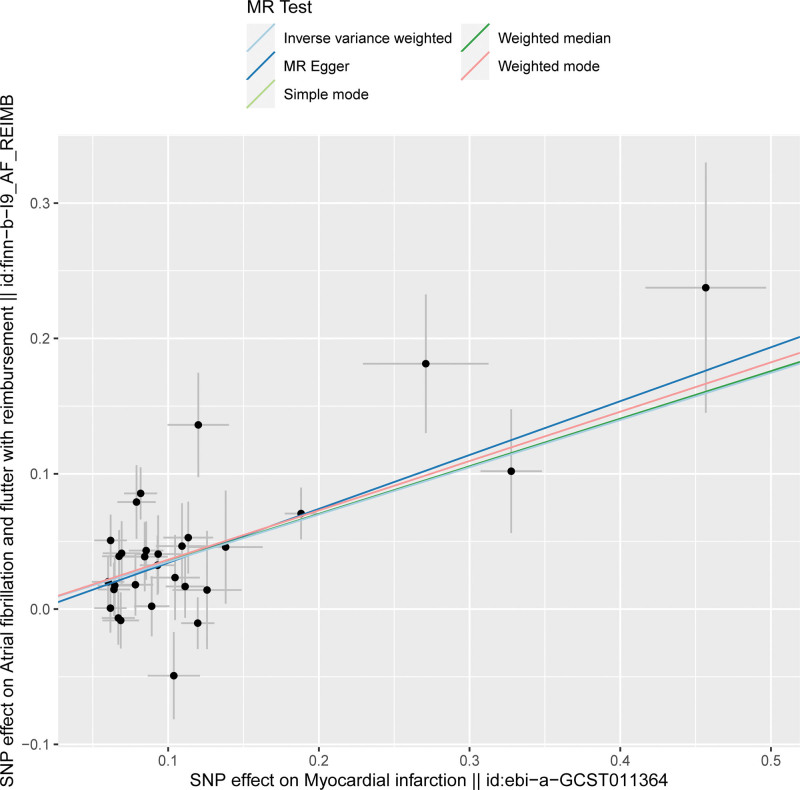
Scatter plot shows the causal relationship between MI and AF based on the individual MI-related IVs. The effect of each MI-related SNP on MI is plotted against the corresponding effect on AF. The slope of each line represents the causal association between MI and AF for each method based on the selected IVs. The light blue line represents the IVW estimate; the bottle green line represents the weighted median estimate; the dark blue line represents the MR-Egger estimate; the red line represents the weighted mode estimate; and the light green line represents the simple mode estimate. AF = atrial fibrillation, IVW = inverse-variance weighted, MI = myocardial infarction, MR = Mendelian randomization.

The SNP signatures related to MI are shown in Table S1, Supplemental Digital Content http://links.lww.com/MD/N837. We selected 30 MI-related SNPs as the IVs (Table S3, Supplemental Digital Content, http://links.lww.com/MD/N837). All the genetic tools related to MI showed genome-wide significance (*P* < 5 × 10^−8^, *F* > 10). None of the outcome-associated SNPs were susceptible to the MI-related IVs. The forest plot (Fig. 3) and the scatter plot (Fig. 4) shows the causal effects of each MI-related genetic variant on AF.

### 3.2. Mendelian randomization results for the causal effects of MI on AF

The causal relationship between MI and AF was estimated using the IVW, MR-Egger, and weighted median regression methods, and the results are shown in Tables 1 and S5, Supplemental Digital Content, http://links.lww.com/MD/N837. Overall, the risk of AF was significantly higher in patients with MI (OR: 1.42; 95% CI: 1.70–1.58, *P* = 6 × 10^−10^).

**Table 1:** Results of the MR analyses for the causal relationship between MI and AF, including the test for heterogeneity.

MR methods	nSNP	OR	95% CI	Beta	SE	*P* value	Heterogeneity test	
							Cochran’s Q statistic	*P* value
MR-Egger	30	1.49	1.15, 1.93	0.398	0.131	.005	50.201	.006
Weighted median	30	1.42	1.24, 1.63	0.352	0.068	<.001		
Inverse-variance weighted	30	1.42	1.27, 1.58	0.349	0.057	<.001	50.506	.008
Simple mode	30	1.44	1.13, 1.84	0.365	0.122	.006		
Weighted mode	30	1.44	1.22, 1.70	0.365	0.089	<.001		
MR-Egger	20	0.98	0.89, 1.06	−0.025	0.045	.580	29.164	.046
Weighted median	20	1.02	0.97, 1.07	0.017	0.024	.485		
Inverse-variance weighted	20	1.05	1.00, 1.09	0.047	0.022	.033	34.464	.016
Simple mode	20	1.01	0.93, 1.10	0.012	0.040	.763		
Weighted mode	20	1.02	0.96, 1.07	0.015	0.029	.603		

AF = atrial fibrillation, CI = confidence interval, MI = myocardial infarction, MR = Mendelian randomization, OR = odds ratio, SE = standard error, SNP = single nucleotide polymorphism.

### 3.3. Mendelian randomization results for the causal effects of AF on MI

**Figure 5.**
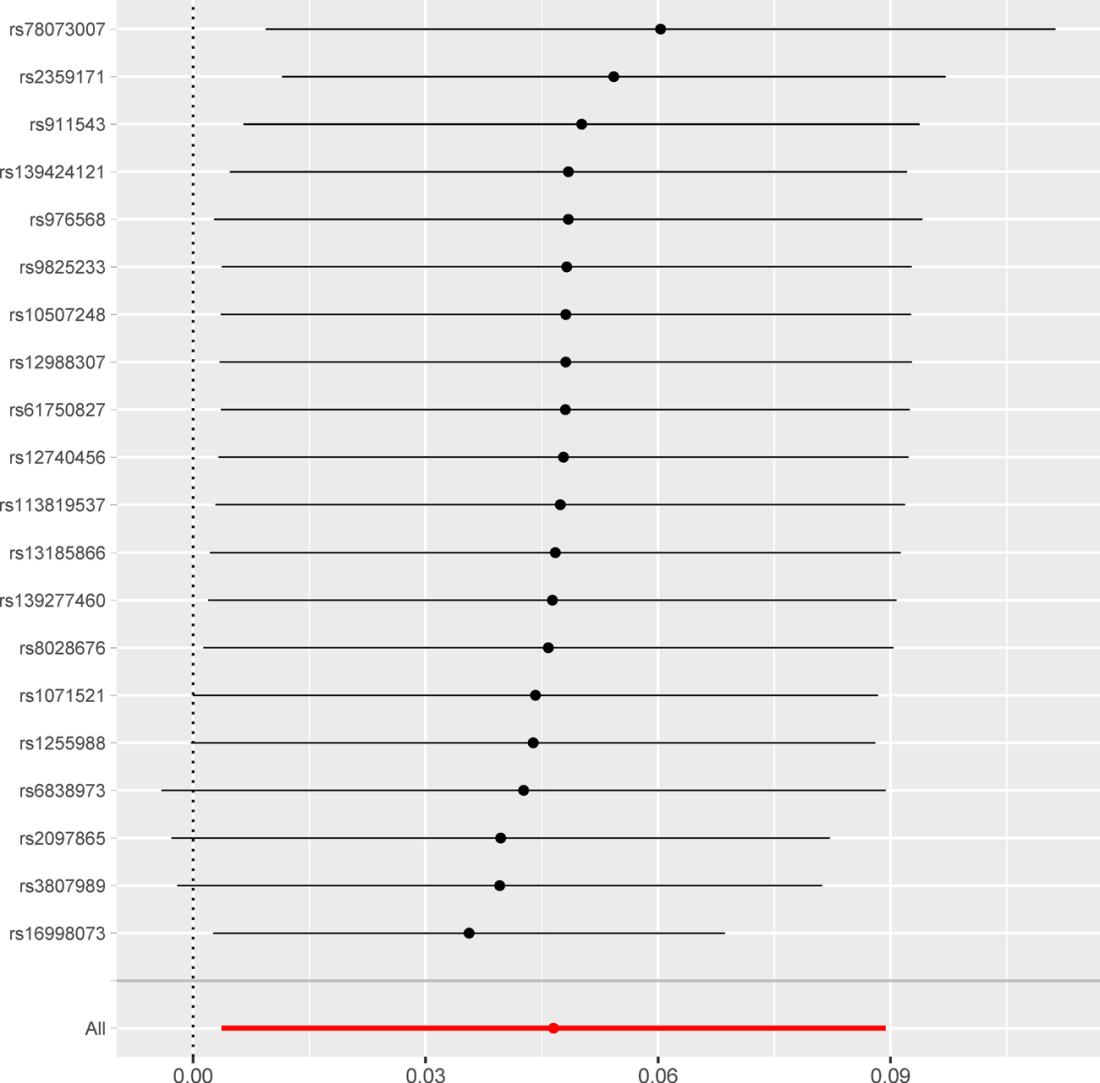
Sensitivity analysis results show the causal effects of AF on MI. AF = atrial fibrillation, MI = myocardial infarction, MR = Mendelian randomization.

**Figure 6.**
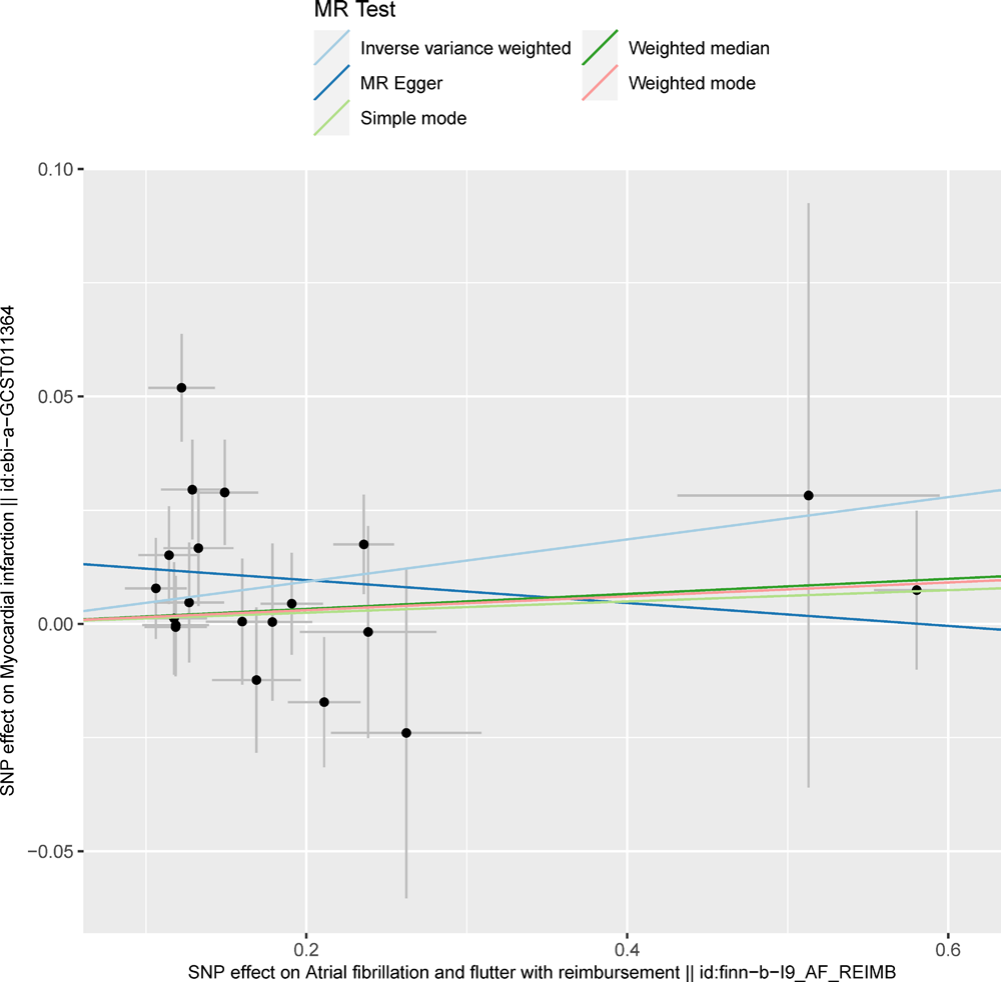
Scatter plot shows the effects of AF-related SNPs on AF compared to their corresponding effects on MI. The slope of each line represents the causal association between AF and MI according to different MR methods. The light blue line represents the IVW estimate; the bottle green line represents the weighted median estimate; the dark blue line represents the MR-Egger estimate; the red line represents the weighted mode estimate; and the light green line represents the simple mode estimate. AF = atrial fibrillation , IVW = inverse-variance weighted, MI = myocardial infarction, MR = Mendelian randomization.

**Figure 7.**
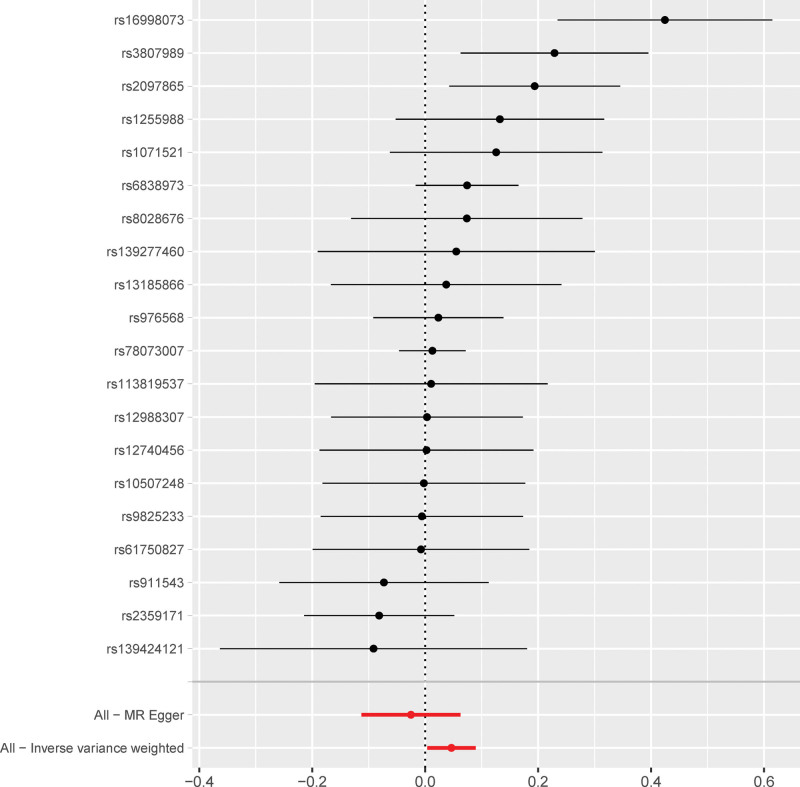
Forest plot shows the causal effects of AF-related SNPs on MI. The black lines represent the effect sizes of the AF-related SNPs. The red lines at the bottom of the plot represent the combined significance MR results for the AF-related SNPs from the MR-Egger test and the IVW method. AF = atrial fibrillation, IVW = inverse-variance weighted, MI = myocardial infarction, MR = Mendelian randomization, SNPs = single nucleotide polymorphisms.

Next, we analyzed whether AF influenced the incidence of MI. Towards this, a bidirectional MR analysis was performed by reversing the exposures and outcomes to determine the effects of a higher genetic risk of AF on MI. We selected 20 SNPs with genome-wide significance for AF (*P* < 5E − 08) that were independently inherited (*r*^2^ < 0.01) without LD, according to the results of the FinnGen project (Tables S2 and S4, Supplemental Digital Content, http://links.lww.com/MD/N837). The data was analyzed using the same MR methods as described previously. The statistical tests used for the bidirectional MR analyses was 2-sided. The results of the MR analyses and the sensitivity analyses showed that the causal effects of AF on MI were statistically significant (Table S6, Supplemental Digital Content, http://links.lww.com/MD/N837). As shown in Table 1, patients with AF showed a significantly higher risk of MI (OR: 1.04; 95% CI: 1.00–1.09, *P* = .03). However, we also observed significant heterogeneity in the results because of the characteristics of the study population (*P* = .01). However, horizontal pleiotropism was not observed in the outcomes (*P* = .08). Leave-one-out analysis showed that the outcome results were stable (Fig. 5). The causal effects of all the AF-associated genetic variants on MI are shown in Figures 6 and 7.

## 4. Discussion

Mendelian randomization method is used to determine causal associations between modifiable exposures and outcomes. However, the reliability of the MR results are dependent on the following 3 core assumptions: genetic variants used as instrument variables are strongly associated with the exposure; IVs are independent of any confounders; and IVs affect the outcome only through their effect on the exposure. In the present study, a bidirectional causal relationship between MI and AF was confirmed using the MR methods by using MI- and AF-related SNPs as the IVs and excluding any potential bias caused by the confounding factors. The specific data regarding MI and AF was extracted from GWAS database and the correlation of all the SNPs that were selected as IVs with the exposure factor was confirmed and effects due to LD were ruled out for all the selected IVs. Horizontal pleiotropy analysis was performed to exclude the SNPs that were related to other confounding factors. GWAS data was then used to determine the relationships between the exposures and the outcomes by analyzing the effect alleles using 5 different MR methods and concurrently testing for any heterogeneity and pleiotropy of the IVs. The results demonstrated the causal effect of MI on AF based on the consistency and monotonicity of the scatter plot, leave-out-one-by-one analysis results, and the distribution of SNPs in the funnel plot. The IVW analysis also showed the causal effect of AF on MI, but the effect was weaker than the causal effect of MI on AF.

The present findings are consistent with those of previous observational studies that have reported a positive association between MI and AF.^[26]^ The causal effects of MI on AF may be due to several plausible reasons. Previous experimental studies have shown that reduced perfusion of the right coronary artery and the left circumflex branch increased the excitability of the atrial myocardium and the conduction velocity, thereby inducing reentry and fibrillation.^[27]^ Furthermore, MI-induced stretching of the atrium prolonged the length of the electrical conduction pathway by increasing the excitability of the atrial myocytes and promoted reentry and fibrillation^[28]^ and MI-induced myocardial fibrosis. MI induces cardiac inflammation through the release of substantial amounts of pro-inflammatory factors, which also play a vital role in the development and maintenance of AF. Besides, the expression of inflammatory factors is also significantly elevated in the non-infarcted and non-ischemic areas. Therefore, the incidence of AF during acute MI may be a marker of widespread inflammation.^[29]^ Moreover, MI is associated with a lower parasympathetic tone, increased sympathetic nerve activity, and secretion of hormones such as B-type natriuretic peptide, all of which may be involved in the development of AF.^[30,31]^

The Atherosclerosis Risk in Communities study showed that patients with AF were associated with a 63% higher risk of acute MI.^[32]^ AF may cause MI through several mechanisms. AF is associated with systemic inflammation, which promotes a pro-thrombotic state that is implicated in the development of MI.^[33]^ Aberrant platelet activation by AF and AF-related risk factors, including hypertension, diabetes, and dyslipidemia, can also promote the development of MI.^[34,35]^ In AF patients with a faster heart rate, increased myocardial oxygen consumption and decreased coronary blood flow causes an imbalance between oxygen supply and demand, which can induce type 2 MI.^[36]^ Stretching of the atrial muscle promotes vasoconstriction by significantly increasing sympathetic nerve activity, stimulating the release of catecholamines from the heart, and activating the adrenergic receptors.^[37]^ However, most of the previous studies are laboratory-based or observational. The present MR study is the first investigation to demonstrate a bidirectional causal relationship between MI and AF. However, the mechanisms underlying these causal relationships remain unclear, and further studies are needed to elucidate the underlying mechanisms.

The present study has several strengths. We used MR to elucidate the bidirectional causal relationship between MI and AF. Our findings can have important clinical implications in the management and treatment of patients with MI or AF.^[38]^ Our data suggests that clinicians treating patients with a history of MI should consider appropriate management strategies to reduce the risk of AF. Conversely, patients with AF should be screened for underlying cardiovascular diseases, including MI. Furthermore, the results of this study suggest the critical importance of improved clinical monitoring strategies to prevent both MI and AF. The evidence of the causal association between MI and AF based on the MR studies is higher than the evidence provided by the observational cohort studies but slightly lower than the evidence provided by the RCTs.^[12]^ However, RCTs are less feasible because of high financial costs. Therefore, MR studies provide robust evidence when RCTs cannot be performed because of ethical or financial considerations. MR studies also provide reliable evidence for the causal relationships between exposures and outcomes by eliminating biases caused by confounding factors and reverse causality.^[39]^ In the MR analysis, selection of the SNPs as IVs can be used to directly infer a causal relationship between the exposure and outcome because the SNPs are randomly distributed in the individuals through genetic inheritance and are not affected by the external environmental or other confounding factors.^[40]^

However, there were several limitations in the present study. Firstly, this MR study was conducted with data from individuals of European descent. Therefore, it is plausible that the interpretations of our findings may not apply to other populations.^[41]^ Secondly, MR analysis is based on the assumption that the genetic variants selected as IVs are not associated with any confounding factors. Therefore, the results could be biased if this assumption were invalid. Thirdly, we only evaluated the relationship between MI and AF, but did not investigate the effects of clinical treatment on either condition. Finally, AF and MI include specific subtypes, such as paroxysmal atrial fibrillation, persistent atrial fibrillation, type 1 MI, and type 2 MI. However, the limitations of the data source prevented us from addressing the causal relationships between these subtypes.

In the future, in-depth investigations are necessary to unravel the mechanisms underlying the causal relationship between MI and AF to identify the potential therapeutic strategies for reducing the risks of both conditions. Furthermore, MR studies should be conducted in diverse populations to determine the bi-directional causal relationship between MI and AF. Finally, investigations of the treatment effects on both MI and AF may provide important insights into the optimal management strategies for both these conditions.

## 5. Conclusion

This MR analysis demonstrated the bidirectional causal relationship between MI and AF in subjects of European ancestry.

## Author contributions

**Conceptualization:** Pei Wang, Zhinong Wang.

**Data curation:** Xuefu Wang.

**Funding acquisition:** Zhinong Wang.

**Investigation:** Jin Rao.

**Methodology:** Yue Yu.

**Project administration:** Zhinong Wang.

**Resources:** Jin Rao.

**Software:** Pengchao Cheng.

**Supervision:** Pei Wang.

**Validation:** Jin Rao, Yue Yu, Pengchao Cheng.

**Visualization:** Jin Rao.

**Writing – original draft:** Jin Rao.

**Writing – review & editing:** Zhinong Wang.

## Supplementary Material


