# Supplementary material for: Relationship between myocardial infarction and atrial fibrillation: A bidirectional Mendelian randomization study
Source: Medicine (Baltimore). 2024 Nov 1;103(44):e40252. doi: 10.1097/MD.0000000000040252 (PMC11537587; doi:10.1097/MD.0000000000040252)
Supplement: Supplementary file 1 [file medi-103-e40252-s001.docx]

Table S1. Signatures of the SNPs associated with MI

| No. | SNP | Gene | Chr. | EA | OA | EAF.MI | EAF.AF | β.MI | β.AF | SE.MI | SE.AF |
| --- | --- | --- | --- | --- | --- | --- | --- | --- | --- | --- | --- |
| 1 | rs10404176 | MAP1S | 19 | G | A | 0.539 | 0.591 | 0.060 | 0.020 | 0.011 | 0.019 |
| 2 | rs10455872 | LPA | 6 | G | A | 0.075 | 0.045 | 0.328 | 0.102 | 0.020 | 0.046 |
| 3 | rs10841443 | LINC02398 | 12 | G | C | 0.659 | 0.754 | 0.065 | 0.017 | 0.011 | 0.022 |
| 4 | rs11591147 | PCSK9 | 1 | T | G | 0.017 | 0.036 | −0.271 | −0.181 | 0.042 | 0.051 |
| 5 | rs11617955 | COL4A1 | 13 | A | T | 0.111 | 0.098 | −0.104 | 0.049 | 0.017 | 0.032 |
| 6 | rs11632963 | / | 15 | A | G | 0.505 | 0.630 | 0.064 | 0.015 | 0.011 | 0.020 |
| 7 | rs117733303 | LPAL2 | 6 | G | A | 0.018 | 0.011 | 0.457 | 0.238 | 0.040 | 0.092 |
| 8 | rs12740374 | CELSR2 | 1 | T | G | 0.222 | 0.216 | −0.111 | −0.017 | 0.013 | 0.023 |
| 9 | rs12906125 | FES | 15 | A | G | 0.321 | 0.268 | 0.085 | 0.043 | 0.012 | 0.022 |
| 10 | rs1384705 | / | 11 | T | C | 0.706 | 0.776 | −0.078 | −0.018 | 0.012 | 0.023 |
| 11 | rs1412444 | LIPA | 10 | T | C | 0.344 | 0.413 | 0.067 | 0.039 | 0.011 | 0.019 |
| 12 | rs1704221 | / | 10 | A | G | 0.107 | 0.102 | −0.109 | −0.047 | 0.017 | 0.032 |
| 13 | rs1964600 | LOC124902609 | 11 | A | G | 0.248 | 0.199 | 0.069 | 0.041 | 0.012 | 0.024 |
| 14 | rs1966248 | LINC01312 | 6 | T | A | 0.309 | 0.234 | −0.089 | −0.002 | 0.012 | 0.022 |
| 15 | rs28451064 | / | 21 | A | G | 0.127 | 0.153 | 0.113 | 0.053 | 0.017 | 0.027 |
| 16 | rs2886722 | / | 2 | G | A | 0.404 | 0.377 | 0.067 | −0.007 | 0.011 | 0.020 |
| 17 | rs2891168 | CDKN2B-AS1 | 9 | G | A | 0.483 | 0.430 | 0.188 | 0.071 | 0.011 | 0.019 |
| 18 | rs2954021 | / | 8 | G | A | 0.507 | 0.538 | −0.062 | −0.001 | 0.011 | 0.018 |
| 19 | rs3127580 | / | 6 | T | C | 0.151 | 0.123 | 0.093 | 0.041 | 0.015 | 0.029 |
| 20 | rs35350651 | ATXN2 | 12 | AC | A | 0.524 | 0.571 | −0.082 | −0.086 | 0.011 | 0.019 |
| 21 | rs376993806 | GPSM1 | 9 | G | A | 0.717 | 0.698 | 0.069 | −0.008 | 0.012 | 0.021 |
| 22 | rs3918226 | NOS3 | 7 | T | C | 0.078 | 0.068 | 0.120 | 0.136 | 0.020 | 0.039 |
| 23 | rs4846384 | MIA3 | 1 | G | C | 0.691 | 0.748 | 0.093 | 0.032 | 0.012 | 0.022 |
| 24 | rs698270 | STAG1 | 3 | G | A | 0.768 | 0.858 | 0.079 | 0.079 | 0.013 | 0.027 |
| 25 | rs7137258 | FLJ12825 | 12 | A | C | 0.062 | 0.050 | 0.126 | 0.014 | 0.023 | 0.044 |
| 26 | rs73015016 | / | 19 | A | G | 0.123 | 0.103 | −0.105 | −0.023 | 0.016 | 0.031 |
| 27 | rs7485656 | SCARB1 | 12 | G | A | 0.151 | 0.170 | 0.084 | 0.039 | 0.015 | 0.026 |
| 28 | rs77870048 | WWP2 | 16 | T | C | 0.050 | 0.056 | −0.138 | −0.046 | 0.025 | 0.042 |
| 29 | rs9349379 | PHACTR1 | 6 | G | A | 0.402 | 0.451 | 0.120 | −0.010 | 0.011 | 0.019 |
| 30 | rs9893777 | / | 17 | T | G | 0.465 | 0.473 | −0.062 | −0.051 | 0.011 | 0.019 |

SNP, single nucleotide polymorphism; MI, myocardial infarction; EA, effect allele; OA, other allele; EAF, effect allele frequency, AF, atrial fibrillation.

Table S2. Signatures of the SNPs associated with AF

| No. | SNP | Gene | Chr. | EA | OA | EAF.AF | EAF.MI | β.AF | β.MI | SE.AF | SE.MI |
| --- | --- | --- | --- | --- | --- | --- | --- | --- | --- | --- | --- |
| 1 | rs10507248 | TBX5 | 12 | T | G | 0.697 | 0.732 | 0.119 | 0.000 | 0.021 | 0.011 |
| 2 | rs1071521 | / | 1 | G | A | 0.746 | 0.770 | −0.133 | −0.017 | 0.022 | 0.013 |
| 3 | rs113819537 | SSPN | 12 | G | C | 0.309 | 0.251 | −0.118 | −0.001 | 0.021 | 0.012 |
| 4 | rs1255988 | SYNE2 | 14 | A | C | 0.523 | 0.508 | −0.114 | −0.015 | 0.019 | 0.011 |
| 5 | rs12740456 | KCNN3 | 1 | A | C | 0.179 | 0.150 | 0.179 | 0.000 | 0.025 | 0.017 |
| 6 | rs12988307 | TTN | 2 | C | T | 0.221 | 0.199 | 0.160 | 0.001 | 0.023 | 0.014 |
| 7 | rs13185866 | PFDN1 | 5 | A | G | 0.256 | 0.204 | 0.127 | 0.005 | 0.022 | 0.013 |
| 8 | rs139277460 | / | 16 | G | C | 0.013 | 0.007 | 0.513 | 0.028 | 0.082 | 0.064 |
| 9 | rs139424121 | / | 1 | A | G | 0.044 | 0.024 | −0.262 | 0.024 | 0.047 | 0.036 |
| 10 | rs16998073 | / | 4 | T | A | 0.310 | 0.288 | 0.122 | 0.052 | 0.021 | 0.012 |
| 11 | rs2097865 | PKD2L2 | 5 | T | C | 0.290 | 0.318 | 0.149 | 0.029 | 0.021 | 0.012 |
| 12 | rs2359171 | ZFHX3 | 16 | A | T | 0.225 | 0.171 | 0.211 | −0.017 | 0.023 | 0.014 |
| 13 | rs3807989 | CAV1 | 7 | G | A | 0.571 | 0.587 | 0.129 | 0.030 | 0.019 | 0.011 |
| 14 | rs61750827 | RHBDL2 | 1 | T | C | 0.053 | 0.059 | 0.239 | −0.002 | 0.043 | 0.023 |
| 15 | rs6838973 | / | 4 | T | C | 0.501 | 0.432 | −0.236 | −0.018 | 0.019 | 0.011 |
| 16 | rs78073007 | / | 4 | T | C | 0.147 | 0.105 | 0.580 | 0.007 | 0.027 | 0.017 |
| 17 | rs8028676 | / | 15 | A | C | 0.419 | 0.400 | 0.106 | 0.008 | 0.019 | 0.011 |
| 18 | rs911543 | SH3PXD2A | 10 | G | A | 0.865 | 0.870 | −0.169 | 0.012 | 0.028 | 0.016 |
| 19 | rs976568 | PITX2 | 4 | T | G | 0.598 | 0.646 | −0.191 | −0.004 | 0.019 | 0.011 |
| 20 | rs9825233 | CAND2 | 3 | T | C | 0.587 | 0.590 | 0.119 | −0.001 | 0.019 | 0.011 |

SNP, single nucleotide polymorphism; AF, atrial fibrillation; EA, effect allele; OA, other allele; EAF, effect allele frequency; MI, myocardial infarction.

Table 3

|  | **SNP** | **effect_allele.exposure** | **other_allele.exposure** | **effect_allele.outcome** | **other_allele.outcome** | **beta.exposure** | **beta.outcome** | **eaf.exposure** | **eaf.outcome** | **remove** | **palindromic** | **ambiguous** | **id.outcome** | **chr** | **pos** | **se.outcome** | **samplesize.outcome** | **pval.outcome** | **outcome** | **originalname.outcome** | **outcome.deprecated** | **mr_keep.outcome** | **data_source.outcome** | **proxy.outcome** | **target_snp.outcome** | **proxy_snp.outcome** | **target_a1.outcome** | **target_a2.outcome** | **proxy_a1.outcome** | **proxy_a2.outcome** | **chr.exposure** | **pos.exposure** | **se.exposure** | **pval.exposure** | **samplesize.exposure** | **id.exposure** | **exposure** | **mr_keep.exposure** | **pval_origin.exposure** | **data_source.exposure** | **action** | **mr_keep** |
| --- | --- | --- | --- | --- | --- | --- | --- | --- | --- | --- | --- | --- | --- | --- | --- | --- | --- | --- | --- | --- | --- | --- | --- | --- | --- | --- | --- | --- | --- | --- | --- | --- | --- | --- | --- | --- | --- | --- | --- | --- | --- | --- |
| 1 | rs10404176 | G | A | G | A | 0.0601737 | 0.0202 | 0.538593 | 0.5905 | FALSE | FALSE | FALSE | finn-b-I9_AF_REIMB | 19 | 17844325 | 0.019421473 | NA | 0.2983 | Atrial fibrillation and flutter with reimbursement \|\| id:finn-b-I9_AF_REIMB | Atrial fibrillation and flutter with reimbursement | Atrial fibrillation and flutter with reimbursement \|\| \|\| | TRUE | igd | TRUE | rs10404176 | rs2287860 | A | G | T | C | 19 | 17829393 | 0.0107876 | 2.40E-08 | 17505 | ebi-a-GCST011364 | Myocardial infarction \|\| id:ebi-a-GCST011364 | TRUE | reported | igd | 2 | TRUE |
| 2 | rs10455872 | G | A | G | A | 0.327656 | 0.1019 | 0.074987 | 0.04486 | FALSE | FALSE | FALSE | finn-b-I9_AF_REIMB | 6 | 161010118 | 0.04572669 | NA | 0.02585 | Atrial fibrillation and flutter with reimbursement \|\| id:finn-b-I9_AF_REIMB | Atrial fibrillation and flutter with reimbursement | Atrial fibrillation and flutter with reimbursement \|\| \|\| | TRUE | igd | NA | NA | NA | NA | NA | NA | NA | 6 | 161010118 | 0.0203866 | 4.00E-58 | 17505 | ebi-a-GCST011364 | Myocardial infarction \|\| id:ebi-a-GCST011364 | TRUE | reported | igd | 2 | TRUE |
| 3 | rs10841443 | G | C | G | C | 0.0645606 | 0.0174 | 0.658908 | 0.7535 | FALSE | TRUE | FALSE | finn-b-I9_AF_REIMB | 12 | 20220033 | 0.022163025 | NA | 0.4324 | Atrial fibrillation and flutter with reimbursement \|\| id:finn-b-I9_AF_REIMB | Atrial fibrillation and flutter with reimbursement | Atrial fibrillation and flutter with reimbursement \|\| \|\| | TRUE | igd | NA | NA | NA | NA | NA | NA | NA | 12 | 20220033 | 0.0114748 | 1.80E-08 | 17505 | ebi-a-GCST011364 | Myocardial infarction \|\| id:ebi-a-GCST011364 | TRUE | reported | igd | 2 | TRUE |
| 4 | rs11591147 | T | G | T | G | -0.270964 | -0.1813 | 0.016784 | 0.03646 | FALSE | FALSE | FALSE | finn-b-I9_AF_REIMB | 1 | 55505647 | 0.051258103 | NA | 0.000404697 | Atrial fibrillation and flutter with reimbursement \|\| id:finn-b-I9_AF_REIMB | Atrial fibrillation and flutter with reimbursement | Atrial fibrillation and flutter with reimbursement \|\| \|\| | TRUE | igd | NA | NA | NA | NA | NA | NA | NA | 1 | 55505647 | 0.0416959 | 8.10E-11 | 17505 | ebi-a-GCST011364 | Myocardial infarction \|\| id:ebi-a-GCST011364 | TRUE | reported | igd | 2 | TRUE |
| 5 | rs11617955 | A | T | A | T | -0.10376 | 0.0492 | 0.111065 | 0.09796 | FALSE | TRUE | FALSE | finn-b-I9_AF_REIMB | 13 | 110818102 | 0.032231832 | NA | 0.1269 | Atrial fibrillation and flutter with reimbursement \|\| id:finn-b-I9_AF_REIMB | Atrial fibrillation and flutter with reimbursement | Atrial fibrillation and flutter with reimbursement \|\| \|\| | TRUE | igd | NA | NA | NA | NA | NA | NA | NA | 13 | 110818102 | 0.017346 | 2.20E-09 | 17505 | ebi-a-GCST011364 | Myocardial infarction \|\| id:ebi-a-GCST011364 | TRUE | reported | igd | 2 | TRUE |
| 6 | rs11632963 | A | G | A | G | 0.0640451 | 0.0145 | 0.505476 | 0.63 | FALSE | FALSE | FALSE | finn-b-I9_AF_REIMB | 15 | 79132644 | 0.019695339 | NA | 0.4616 | Atrial fibrillation and flutter with reimbursement \|\| id:finn-b-I9_AF_REIMB | Atrial fibrillation and flutter with reimbursement | Atrial fibrillation and flutter with reimbursement \|\| \|\| | TRUE | igd | NA | NA | NA | NA | NA | NA | NA | 15 | 79132644 | 0.0107396 | 2.50E-09 | 17505 | ebi-a-GCST011364 | Myocardial infarction \|\| id:ebi-a-GCST011364 | TRUE | reported | igd | 2 | TRUE |
| 7 | rs117733303 | G | A | G | A | 0.456742 | 0.2375 | 0.018334 | 0.01105 | FALSE | FALSE | FALSE | finn-b-I9_AF_REIMB | 6 | 160922870 | 0.092486113 | NA | 0.0102301 | Atrial fibrillation and flutter with reimbursement \|\| id:finn-b-I9_AF_REIMB | Atrial fibrillation and flutter with reimbursement | Atrial fibrillation and flutter with reimbursement \|\| \|\| | TRUE | igd | NA | NA | NA | NA | NA | NA | NA | 6 | 160922870 | 0.0400419 | 3.90E-30 | 17505 | ebi-a-GCST011364 | Myocardial infarction \|\| id:ebi-a-GCST011364 | TRUE | reported | igd | 2 | TRUE |
| 8 | rs12740374 | T | G | T | G | -0.11127 | -0.0167 | 0.222239 | 0.2158 | FALSE | FALSE | FALSE | finn-b-I9_AF_REIMB | 1 | 109817590 | 0.023203604 | NA | 0.4717 | Atrial fibrillation and flutter with reimbursement \|\| id:finn-b-I9_AF_REIMB | Atrial fibrillation and flutter with reimbursement | Atrial fibrillation and flutter with reimbursement \|\| \|\| | TRUE | igd | NA | NA | NA | NA | NA | NA | NA | 1 | 109817590 | 0.0128803 | 5.70E-18 | 17505 | ebi-a-GCST011364 | Myocardial infarction \|\| id:ebi-a-GCST011364 | TRUE | reported | igd | 2 | TRUE |
| 9 | rs12906125 | A | G | A | G | 0.0854004 | 0.0432 | 0.321354 | 0.2684 | FALSE | FALSE | FALSE | finn-b-I9_AF_REIMB | 15 | 91427612 | 0.021586974 | NA | 0.0453701 | Atrial fibrillation and flutter with reimbursement \|\| id:finn-b-I9_AF_REIMB | Atrial fibrillation and flutter with reimbursement | Atrial fibrillation and flutter with reimbursement \|\| \|\| | TRUE | igd | NA | NA | NA | NA | NA | NA | NA | 15 | 91427612 | 0.0115252 | 1.30E-13 | 17505 | ebi-a-GCST011364 | Myocardial infarction \|\| id:ebi-a-GCST011364 | TRUE | reported | igd | 2 | TRUE |
| 10 | rs1384705 | T | C | T | C | -0.0782861 | -0.018 | 0.706091 | 0.7761 | FALSE | FALSE | FALSE | finn-b-I9_AF_REIMB | 11 | 103696851 | 0.022788378 | NA | 0.4296 | Atrial fibrillation and flutter with reimbursement \|\| id:finn-b-I9_AF_REIMB | Atrial fibrillation and flutter with reimbursement | Atrial fibrillation and flutter with reimbursement \|\| \|\| | TRUE | igd | NA | NA | NA | NA | NA | NA | NA | 11 | 103696851 | 0.0117986 | 3.20E-11 | 17505 | ebi-a-GCST011364 | Myocardial infarction \|\| id:ebi-a-GCST011364 | TRUE | reported | igd | 2 | TRUE |
| 11 | rs1412444 | T | C | T | C | 0.0674457 | 0.039 | 0.344095 | 0.4126 | FALSE | FALSE | FALSE | finn-b-I9_AF_REIMB | 10 | 91002927 | 0.019310304 | NA | 0.04342 | Atrial fibrillation and flutter with reimbursement \|\| id:finn-b-I9_AF_REIMB | Atrial fibrillation and flutter with reimbursement | Atrial fibrillation and flutter with reimbursement \|\| \|\| | TRUE | igd | NA | NA | NA | NA | NA | NA | NA | 10 | 91002927 | 0.011283 | 2.30E-09 | 17505 | ebi-a-GCST011364 | Myocardial infarction \|\| id:ebi-a-GCST011364 | TRUE | reported | igd | 2 | TRUE |
| 12 | rs1704221 | A | G | A | G | -0.109198 | -0.0465 | 0.107122 | 0.1022 | FALSE | FALSE | FALSE | finn-b-I9_AF_REIMB | 10 | 44710930 | 0.031731012 | NA | 0.1428 | Atrial fibrillation and flutter with reimbursement \|\| id:finn-b-I9_AF_REIMB | Atrial fibrillation and flutter with reimbursement | Atrial fibrillation and flutter with reimbursement \|\| \|\| | TRUE | igd | NA | NA | NA | NA | NA | NA | NA | 10 | 44710930 | 0.0174122 | 3.60E-10 | 17505 | ebi-a-GCST011364 | Myocardial infarction \|\| id:ebi-a-GCST011364 | TRUE | reported | igd | 2 | TRUE |
| 13 | rs1964600 | A | G | A | G | 0.0691276 | 0.0412 | 0.247629 | 0.1989 | FALSE | FALSE | FALSE | finn-b-I9_AF_REIMB | 11 | 1680664 | 0.023805043 | NA | 0.0835007 | Atrial fibrillation and flutter with reimbursement \|\| id:finn-b-I9_AF_REIMB | Atrial fibrillation and flutter with reimbursement | Atrial fibrillation and flutter with reimbursement \|\| \|\| | TRUE | igd | NA | NA | NA | NA | NA | NA | NA | 11 | 1680664 | 0.0124958 | 3.20E-08 | 17505 | ebi-a-GCST011364 | Myocardial infarction \|\| id:ebi-a-GCST011364 | TRUE | reported | igd | 2 | TRUE |
| 14 | rs1966248 | T | A | T | A | -0.0890783 | -0.0021 | 0.309381 | 0.2343 | FALSE | TRUE | FALSE | finn-b-I9_AF_REIMB | 6 | 134159622 | 0.022278023 | NA | 0.9249 | Atrial fibrillation and flutter with reimbursement \|\| id:finn-b-I9_AF_REIMB | Atrial fibrillation and flutter with reimbursement | Atrial fibrillation and flutter with reimbursement \|\| \|\| | TRUE | igd | NA | NA | NA | NA | NA | NA | NA | 6 | 134159622 | 0.0117383 | 3.20E-14 | 17505 | ebi-a-GCST011364 | Myocardial infarction \|\| id:ebi-a-GCST011364 | TRUE | reported | igd | 2 | TRUE |
| 15 | rs28451064 | A | G | A | G | 0.113339 | 0.0528 | 0.126529 | 0.1529 | FALSE | FALSE | FALSE | finn-b-I9_AF_REIMB | 21 | 35593827 | 0.026545894 | NA | 0.0467003 | Atrial fibrillation and flutter with reimbursement \|\| id:finn-b-I9_AF_REIMB | Atrial fibrillation and flutter with reimbursement | Atrial fibrillation and flutter with reimbursement \|\| \|\| | TRUE | igd | NA | NA | NA | NA | NA | NA | NA | 21 | 35593827 | 0.0165161 | 6.80E-12 | 17505 | ebi-a-GCST011364 | Myocardial infarction \|\| id:ebi-a-GCST011364 | TRUE | reported | igd | 2 | TRUE |
| 16 | rs2886722 | G | A | G | A | 0.0669274 | -0.0066 | 0.40358 | 0.3771 | FALSE | FALSE | FALSE | finn-b-I9_AF_REIMB | 2 | 85742297 | 0.019738584 | NA | 0.7381 | Atrial fibrillation and flutter with reimbursement \|\| id:finn-b-I9_AF_REIMB | Atrial fibrillation and flutter with reimbursement | Atrial fibrillation and flutter with reimbursement \|\| \|\| | TRUE | igd | NA | NA | NA | NA | NA | NA | NA | 2 | 85742297 | 0.0109925 | 1.10E-09 | 17505 | ebi-a-GCST011364 | Myocardial infarction \|\| id:ebi-a-GCST011364 | TRUE | reported | igd | 2 | TRUE |
| 17 | rs2891168 | G | A | G | A | 0.188202 | 0.0706 | 0.483196 | 0.4296 | FALSE | FALSE | FALSE | finn-b-I9_AF_REIMB | 9 | 22098619 | 0.019195957 | NA | 0.000235201 | Atrial fibrillation and flutter with reimbursement \|\| id:finn-b-I9_AF_REIMB | Atrial fibrillation and flutter with reimbursement | Atrial fibrillation and flutter with reimbursement \|\| \|\| | TRUE | igd | NA | NA | NA | NA | NA | NA | NA | 9 | 22098619 | 0.0107654 | 2.00E-68 | 17505 | ebi-a-GCST011364 | Myocardial infarction \|\| id:ebi-a-GCST011364 | TRUE | reported | igd | 2 | TRUE |
| 18 | rs2954021 | G | A | G | A | -0.0617065 | -7.00E-04 | 0.506775 | 0.5378 | FALSE | FALSE | FALSE | finn-b-I9_AF_REIMB | 8 | 126482077 | 0.018129235 | NA | 0.9692 | Atrial fibrillation and flutter with reimbursement \|\| id:finn-b-I9_AF_REIMB | Atrial fibrillation and flutter with reimbursement | Atrial fibrillation and flutter with reimbursement \|\| \|\| | TRUE | igd | NA | NA | NA | NA | NA | NA | NA | 8 | 126482077 | 0.0107304 | 8.90E-09 | 17505 | ebi-a-GCST011364 | Myocardial infarction \|\| id:ebi-a-GCST011364 | TRUE | reported | igd | 2 | TRUE |
| 19 | rs3127580 | T | C | T | C | 0.093356 | 0.0406 | 0.150844 | 0.1232 | FALSE | FALSE | FALSE | finn-b-I9_AF_REIMB | 6 | 160710851 | 0.028694758 | NA | 0.1571 | Atrial fibrillation and flutter with reimbursement \|\| id:finn-b-I9_AF_REIMB | Atrial fibrillation and flutter with reimbursement | Atrial fibrillation and flutter with reimbursement \|\| \|\| | TRUE | igd | NA | NA | NA | NA | NA | NA | NA | 6 | 160710851 | 0.015 | 4.90E-10 | 17505 | ebi-a-GCST011364 | Myocardial infarction \|\| id:ebi-a-GCST011364 | TRUE | reported | igd | 2 | TRUE |
| 20 | rs35350651 | AC | A | AC | A | -0.0817516 | -0.0855 | 0.523593 | 0.5712 | FALSE | FALSE | FALSE | finn-b-I9_AF_REIMB | 12 | 111907431 | 0.019283107 | NA | 9.25E-06 | Atrial fibrillation and flutter with reimbursement \|\| id:finn-b-I9_AF_REIMB | Atrial fibrillation and flutter with reimbursement | Atrial fibrillation and flutter with reimbursement \|\| \|\| | TRUE | igd | NA | NA | NA | NA | NA | NA | NA | 12 | 111907431 | 0.0109192 | 7.00E-14 | 17505 | ebi-a-GCST011364 | Myocardial infarction \|\| id:ebi-a-GCST011364 | TRUE | reported | igd | 2 | TRUE |
| 21 | rs376993806 | G | A | G | A | 0.068534 | -0.0084 | 0.716864 | 0.6983 | FALSE | FALSE | FALSE | finn-b-I9_AF_REIMB | 9 | 139248082 | 0.020735342 | NA | 0.6854 | Atrial fibrillation and flutter with reimbursement \|\| id:finn-b-I9_AF_REIMB | Atrial fibrillation and flutter with reimbursement | Atrial fibrillation and flutter with reimbursement \|\| \|\| | TRUE | igd | TRUE | rs376993806 | rs28642213 | A | G | A | G | 9 | 139246588 | 0.0121549 | 1.70E-08 | 17505 | ebi-a-GCST011364 | Myocardial infarction \|\| id:ebi-a-GCST011364 | TRUE | reported | igd | 2 | TRUE |
| 22 | rs3918226 | T | C | T | C | 0.12 | 0.1361 | 0.077561 | 0.06813 | FALSE | FALSE | FALSE | finn-b-I9_AF_REIMB | 7 | 150690176 | 0.038560406 | NA | 0.000416303 | Atrial fibrillation and flutter with reimbursement \|\| id:finn-b-I9_AF_REIMB | Atrial fibrillation and flutter with reimbursement | Atrial fibrillation and flutter with reimbursement \|\| \|\| | TRUE | igd | NA | NA | NA | NA | NA | NA | NA | 7 | 150690176 | 0.0203657 | 3.80E-09 | 17505 | ebi-a-GCST011364 | Myocardial infarction \|\| id:ebi-a-GCST011364 | TRUE | reported | igd | 2 | TRUE |
| 23 | rs4846384 | G | C | G | C | 0.0929653 | 0.0324 | 0.691031 | 0.7476 | FALSE | TRUE | FALSE | finn-b-I9_AF_REIMB | 1 | 222797614 | 0.021887832 | NA | 0.1388 | Atrial fibrillation and flutter with reimbursement \|\| id:finn-b-I9_AF_REIMB | Atrial fibrillation and flutter with reimbursement | Atrial fibrillation and flutter with reimbursement \|\| \|\| | TRUE | igd | NA | NA | NA | NA | NA | NA | NA | 1 | 222797614 | 0.011788 | 3.10E-15 | 17505 | ebi-a-GCST011364 | Myocardial infarction \|\| id:ebi-a-GCST011364 | TRUE | reported | igd | 2 | TRUE |
| 24 | rs698270 | G | A | G | A | 0.0790092 | 0.0791 | 0.767827 | 0.8579 | FALSE | FALSE | FALSE | finn-b-I9_AF_REIMB | 3 | 136109512 | 0.027213285 | NA | 0.003653 | Atrial fibrillation and flutter with reimbursement \|\| id:finn-b-I9_AF_REIMB | Atrial fibrillation and flutter with reimbursement | Atrial fibrillation and flutter with reimbursement \|\| \|\| | TRUE | igd | NA | NA | NA | NA | NA | NA | NA | 3 | 136109512 | 0.012689 | 4.80E-10 | 17505 | ebi-a-GCST011364 | Myocardial infarction \|\| id:ebi-a-GCST011364 | TRUE | reported | igd | 2 | TRUE |
| 25 | rs7137258 | A | C | A | C | 0.125799 | 0.0141 | 0.061975 | 0.05035 | FALSE | FALSE | FALSE | finn-b-I9_AF_REIMB | 12 | 54512164 | 0.043671774 | NA | 0.746799 | Atrial fibrillation and flutter with reimbursement \|\| id:finn-b-I9_AF_REIMB | Atrial fibrillation and flutter with reimbursement | Atrial fibrillation and flutter with reimbursement \|\| \|\| | TRUE | igd | NA | NA | NA | NA | NA | NA | NA | 12 | 54512164 | 0.0228806 | 3.80E-08 | 17505 | ebi-a-GCST011364 | Myocardial infarction \|\| id:ebi-a-GCST011364 | TRUE | reported | igd | 2 | TRUE |
| 26 | rs73015016 | A | G | A | G | -0.104569 | -0.0233 | 0.123328 | 0.1032 | FALSE | FALSE | FALSE | finn-b-I9_AF_REIMB | 19 | 11191300 | 0.031416477 | NA | 0.4583 | Atrial fibrillation and flutter with reimbursement \|\| id:finn-b-I9_AF_REIMB | Atrial fibrillation and flutter with reimbursement | Atrial fibrillation and flutter with reimbursement \|\| \|\| | TRUE | igd | NA | NA | NA | NA | NA | NA | NA | 19 | 11191300 | 0.0163826 | 1.70E-10 | 17505 | ebi-a-GCST011364 | Myocardial infarction \|\| id:ebi-a-GCST011364 | TRUE | reported | igd | 2 | TRUE |
| 27 | rs7485656 | G | A | G | A | 0.0844413 | 0.0387 | 0.151356 | 0.1697 | FALSE | FALSE | FALSE | finn-b-I9_AF_REIMB | 12 | 125315647 | 0.025599639 | NA | 0.1306 | Atrial fibrillation and flutter with reimbursement \|\| id:finn-b-I9_AF_REIMB | Atrial fibrillation and flutter with reimbursement | Atrial fibrillation and flutter with reimbursement \|\| \|\| | TRUE | igd | NA | NA | NA | NA | NA | NA | NA | 12 | 125315647 | 0.0152378 | 3.00E-08 | 17505 | ebi-a-GCST011364 | Myocardial infarction \|\| id:ebi-a-GCST011364 | TRUE | reported | igd | 2 | TRUE |
| 28 | rs77870048 | T | C | T | C | -0.138162 | -0.0458 | 0.050246 | 0.05585 | FALSE | FALSE | FALSE | finn-b-I9_AF_REIMB | 16 | 69965021 | 0.041816316 | NA | 0.2734 | Atrial fibrillation and flutter with reimbursement \|\| id:finn-b-I9_AF_REIMB | Atrial fibrillation and flutter with reimbursement | Atrial fibrillation and flutter with reimbursement \|\| \|\| | TRUE | igd | NA | NA | NA | NA | NA | NA | NA | 16 | 69965021 | 0.0245663 | 1.90E-08 | 17505 | ebi-a-GCST011364 | Myocardial infarction \|\| id:ebi-a-GCST011364 | TRUE | reported | igd | 2 | TRUE |
| 29 | rs9349379 | G | A | G | A | 0.11964 | -0.0104 | 0.401524 | 0.4507 | FALSE | FALSE | FALSE | finn-b-I9_AF_REIMB | 6 | 12903957 | 0.019156451 | NA | 0.5872 | Atrial fibrillation and flutter with reimbursement \|\| id:finn-b-I9_AF_REIMB | Atrial fibrillation and flutter with reimbursement | Atrial fibrillation and flutter with reimbursement \|\| \|\| | TRUE | igd | NA | NA | NA | NA | NA | NA | NA | 6 | 12903957 | 0.010997 | 1.40E-27 | 17505 | ebi-a-GCST011364 | Myocardial infarction \|\| id:ebi-a-GCST011364 | TRUE | reported | igd | 2 | TRUE |
| 30 | rs9893777 | T | G | T | G | -0.0618277 | -0.0507 | 0.465205 | 0.4725 | FALSE | FALSE | FALSE | finn-b-I9_AF_REIMB | 17 | 47336686 | 0.019114399 | NA | 0.00799098 | Atrial fibrillation and flutter with reimbursement \|\| id:finn-b-I9_AF_REIMB | Atrial fibrillation and flutter with reimbursement | Atrial fibrillation and flutter with reimbursement \|\| \|\| | TRUE | igd | NA | NA | NA | NA | NA | NA | NA | 17 | 47336686 | 0.0108146 | 1.10E-08 | 17505 | ebi-a-GCST011364 | Myocardial infarction \|\| id:ebi-a-GCST011364 | TRUE | reported | igd | 2 | TRUE |

Table 4

|  | **SNP** | **effect_allele.exposure** | **other_allele.exposure** | **effect_allele.outcome** | **other_allele.outcome** | **beta.exposure** | **beta.outcome** | **eaf.exposure** | **eaf.outcome** | **remove** | **palindromic** | **ambiguous** | **id.outcome** | **chr** | **pos** | **se.outcome** | **samplesize.outcome** | **pval.outcome** | **outcome** | **originalname.outcome** | **outcome.deprecated** | **mr_keep.outcome** | **data_source.outcome** | **se.exposure** | **pos.exposure** | **samplesize.exposure** | **pval.exposure** | **chr.exposure** | **id.exposure** | **exposure** | **mr_keep.exposure** | **pval_origin.exposure** | **data_source.exposure** | **action** | **mr_keep** |
| --- | --- | --- | --- | --- | --- | --- | --- | --- | --- | --- | --- | --- | --- | --- | --- | --- | --- | --- | --- | --- | --- | --- | --- | --- | --- | --- | --- | --- | --- | --- | --- | --- | --- | --- | --- |
| 1 | rs10507248 | T | G | T | G | 0.1186 | -0.000272272 | 0.697 | 0.731572 | FALSE | FALSE | FALSE | ebi-a-GCST011364 | 12 | 114797093 | 0.010860944 | 17505 | 0.98 | Myocardial infarction \|\| id:ebi-a-GCST011364 | Myocardial infarction | Myocardial infarction \|\| \|\| | TRUE | igd | 0.0208 | 114797093 | NA | 1.18E-08 | 12 | finn-b-I9_AF_REIMB | Atrial fibrillation and flutter with reimbursement \|\| id:finn-b-I9_AF_REIMB | TRUE | reported | igd | 2 | TRUE |
| 2 | rs1071521 | G | A | G | A | -0.1327 | -0.016682 | 0.7462 | 0.769728 | FALSE | FALSE | FALSE | ebi-a-GCST011364 | 1 | 170356917 | 0.012728724 | 17505 | 0.19 | Myocardial infarction \|\| id:ebi-a-GCST011364 | Myocardial infarction | Myocardial infarction \|\| \|\| | TRUE | igd | 0.0218 | 170356917 | NA | 1.07E-09 | 1 | finn-b-I9_AF_REIMB | Atrial fibrillation and flutter with reimbursement \|\| id:finn-b-I9_AF_REIMB | TRUE | reported | igd | 2 | TRUE |
| 3 | rs113819537 | G | C | G | C | -0.1175 | -0.00124112 | 0.3093 | 0.250988 | FALSE | TRUE | FALSE | ebi-a-GCST011364 | 12 | 26348429 | 0.012357603 | 17505 | 0.92 | Myocardial infarction \|\| id:ebi-a-GCST011364 | Myocardial infarction | Myocardial infarction \|\| \|\| | TRUE | igd | 0.0206 | 26348429 | NA | 1.27E-08 | 12 | finn-b-I9_AF_REIMB | Atrial fibrillation and flutter with reimbursement \|\| id:finn-b-I9_AF_REIMB | TRUE | reported | igd | 2 | TRUE |
| 4 | rs1255988 | A | C | A | C | -0.1144 | -0.0151362 | 0.5225 | 0.50813 | FALSE | FALSE | FALSE | ebi-a-GCST011364 | 14 | 64657684 | 0.010772547 | 17505 | 0.16 | Myocardial infarction \|\| id:ebi-a-GCST011364 | Myocardial infarction | Myocardial infarction \|\| \|\| | TRUE | igd | 0.0191 | 64657684 | NA | 2.25E-09 | 14 | finn-b-I9_AF_REIMB | Atrial fibrillation and flutter with reimbursement \|\| id:finn-b-I9_AF_REIMB | TRUE | reported | igd | 2 | TRUE |
| 5 | rs12740456 | A | C | A | C | 0.1788 | 0.000432788 | 0.1788 | 0.150021 | FALSE | FALSE | FALSE | ebi-a-GCST011364 | 1 | 154814197 | 0.017263935 | 17505 | 0.98 | Myocardial infarction \|\| id:ebi-a-GCST011364 | Myocardial infarction | Myocardial infarction \|\| \|\| | TRUE | igd | 0.0248 | 154814197 | NA | 5.52E-13 | 1 | finn-b-I9_AF_REIMB | Atrial fibrillation and flutter with reimbursement \|\| id:finn-b-I9_AF_REIMB | TRUE | reported | igd | 2 | TRUE |
| 6 | rs12988307 | C | T | C | T | 0.16 | 0.000521392 | 0.2212 | 0.199403 | FALSE | FALSE | FALSE | ebi-a-GCST011364 | 2 | 179490478 | 0.013863753 | 17505 | 0.97 | Myocardial infarction \|\| id:ebi-a-GCST011364 | Myocardial infarction | Myocardial infarction \|\| \|\| | TRUE | igd | 0.0227 | 179490478 | NA | 1.73E-12 | 2 | finn-b-I9_AF_REIMB | Atrial fibrillation and flutter with reimbursement \|\| id:finn-b-I9_AF_REIMB | TRUE | reported | igd | 2 | TRUE |
| 7 | rs13185866 | A | G | A | G | 0.1269 | 0.00473329 | 0.2555 | 0.204105 | FALSE | FALSE | FALSE | ebi-a-GCST011364 | 5 | 139681897 | 0.013204509 | 17505 | 0.719999 | Myocardial infarction \|\| id:ebi-a-GCST011364 | Myocardial infarction | Myocardial infarction \|\| \|\| | TRUE | igd | 0.0218 | 139681897 | NA | 5.87E-09 | 5 | finn-b-I9_AF_REIMB | Atrial fibrillation and flutter with reimbursement \|\| id:finn-b-I9_AF_REIMB | TRUE | reported | igd | 2 | TRUE |
| 8 | rs139277460 | G | C | G | C | 0.513 | 0.0282483 | 0.01333 | 0.007411 | FALSE | TRUE | FALSE | ebi-a-GCST011364 | 16 | 1982336 | 0.064213354 | 17505 | 0.66 | Myocardial infarction \|\| id:ebi-a-GCST011364 | Myocardial infarction | Myocardial infarction \|\| \|\| | TRUE | igd | 0.0817 | 1982336 | NA | 3.37E-10 | 16 | finn-b-I9_AF_REIMB | Atrial fibrillation and flutter with reimbursement \|\| id:finn-b-I9_AF_REIMB | TRUE | reported | igd | 2 | TRUE |
| 9 | rs139424121 | A | G | A | G | -0.2623 | 0.0239625 | 0.04449 | 0.023561 | FALSE | FALSE | FALSE | ebi-a-GCST011364 | 1 | 10882452 | 0.03637087 | 17505 | 0.51 | Myocardial infarction \|\| id:ebi-a-GCST011364 | Myocardial infarction | Myocardial infarction \|\| \|\| | TRUE | igd | 0.0469 | 10882452 | NA | 2.29E-08 | 1 | finn-b-I9_AF_REIMB | Atrial fibrillation and flutter with reimbursement \|\| id:finn-b-I9_AF_REIMB | TRUE | reported | igd | 2 | TRUE |
| 10 | rs16998073 | T | A | T | A | 0.1222 | 0.0518708 | 0.3104 | 0.287776 | FALSE | TRUE | FALSE | ebi-a-GCST011364 | 4 | 81184341 | 0.011849174 | 17505 | 1.20E-05 | Myocardial infarction \|\| id:ebi-a-GCST011364 | Myocardial infarction | Myocardial infarction \|\| \|\| | TRUE | igd | 0.0207 | 81184341 | NA | 3.44E-09 | 4 | finn-b-I9_AF_REIMB | Atrial fibrillation and flutter with reimbursement \|\| id:finn-b-I9_AF_REIMB | TRUE | reported | igd | 2 | TRUE |
| 11 | rs2097865 | T | C | T | C | 0.1491 | 0.0289138 | 0.2901 | 0.317823 | FALSE | FALSE | FALSE | ebi-a-GCST011364 | 5 | 137245067 | 0.011509609 | 17505 | 0.012 | Myocardial infarction \|\| id:ebi-a-GCST011364 | Myocardial infarction | Myocardial infarction \|\| \|\| | TRUE | igd | 0.0209 | 137245067 | NA | 1.08E-12 | 5 | finn-b-I9_AF_REIMB | Atrial fibrillation and flutter with reimbursement \|\| id:finn-b-I9_AF_REIMB | TRUE | reported | igd | 2 | TRUE |
| 12 | rs2359171 | A | T | A | T | 0.2111 | -0.0171895 | 0.2249 | 0.171328 | FALSE | TRUE | FALSE | ebi-a-GCST011364 | 16 | 73053022 | 0.014320301 | 17505 | 0.23 | Myocardial infarction \|\| id:ebi-a-GCST011364 | Myocardial infarction | Myocardial infarction \|\| \|\| | TRUE | igd | 0.0226 | 73053022 | NA | 8.95E-21 | 16 | finn-b-I9_AF_REIMB | Atrial fibrillation and flutter with reimbursement \|\| id:finn-b-I9_AF_REIMB | TRUE | reported | igd | 2 | TRUE |
| 13 | rs3807989 | G | A | G | A | 0.1289 | 0.0295203 | 0.5705 | 0.586599 | FALSE | FALSE | FALSE | ebi-a-GCST011364 | 7 | 116186241 | 0.01092684 | 17505 | 0.00690001 | Myocardial infarction \|\| id:ebi-a-GCST011364 | Myocardial infarction | Myocardial infarction \|\| \|\| | TRUE | igd | 0.0194 | 116186241 | NA | 2.75E-11 | 7 | finn-b-I9_AF_REIMB | Atrial fibrillation and flutter with reimbursement \|\| id:finn-b-I9_AF_REIMB | TRUE | reported | igd | 2 | TRUE |
| 14 | rs61750827 | T | C | T | C | 0.2385 | -0.00175544 | 0.05325 | 0.059091 | FALSE | FALSE | FALSE | ebi-a-GCST011364 | 1 | 39384625 | 0.023321951 | 17505 | 0.94 | Myocardial infarction \|\| id:ebi-a-GCST011364 | Myocardial infarction | Myocardial infarction \|\| \|\| | TRUE | igd | 0.0425 | 39384625 | NA | 2.07E-08 | 1 | finn-b-I9_AF_REIMB | Atrial fibrillation and flutter with reimbursement \|\| id:finn-b-I9_AF_REIMB | TRUE | reported | igd | 2 | TRUE |
| 15 | rs6838973 | T | C | T | C | -0.2358 | -0.0175073 | 0.5009 | 0.432139 | FALSE | FALSE | FALSE | ebi-a-GCST011364 | 4 | 111765495 | 0.010954433 | 17505 | 0.11 | Myocardial infarction \|\| id:ebi-a-GCST011364 | Myocardial infarction | Myocardial infarction \|\| \|\| | TRUE | igd | 0.0189 | 111765495 | NA | 1.42E-35 | 4 | finn-b-I9_AF_REIMB | Atrial fibrillation and flutter with reimbursement \|\| id:finn-b-I9_AF_REIMB | TRUE | reported | igd | 2 | TRUE |
| 16 | rs78073007 | T | C | T | C | 0.5804 | 0.00744495 | 0.1474 | 0.105197 | FALSE | FALSE | FALSE | ebi-a-GCST011364 | 4 | 111663933 | 0.017470339 | 17505 | 0.67 | Myocardial infarction \|\| id:ebi-a-GCST011364 | Myocardial infarction | Myocardial infarction \|\| \|\| | TRUE | igd | 0.0265 | 111663933 | NA | 5.56E-106 | 4 | finn-b-I9_AF_REIMB | Atrial fibrillation and flutter with reimbursement \|\| id:finn-b-I9_AF_REIMB | TRUE | reported | igd | 2 | TRUE |
| 17 | rs8028676 | A | C | A | C | 0.1062 | 0.00783756 | 0.4194 | 0.400271 | FALSE | FALSE | FALSE | ebi-a-GCST011364 | 15 | 80667367 | 0.011096604 | 17505 | 0.48 | Myocardial infarction \|\| id:ebi-a-GCST011364 | Myocardial infarction | Myocardial infarction \|\| \|\| | TRUE | igd | 0.0192 | 80667367 | NA | 3.23E-08 | 15 | finn-b-I9_AF_REIMB | Atrial fibrillation and flutter with reimbursement \|\| id:finn-b-I9_AF_REIMB | TRUE | reported | igd | 2 | TRUE |
| 18 | rs911543 | G | A | G | A | -0.1689 | 0.012326 | 0.8651 | 0.869661 | FALSE | FALSE | FALSE | ebi-a-GCST011364 | 10 | 105546851 | 0.015962326 | 17505 | 0.44 | Myocardial infarction \|\| id:ebi-a-GCST011364 | Myocardial infarction | Myocardial infarction \|\| \|\| | TRUE | igd | 0.0276 | 105546851 | NA | 1.01E-09 | 10 | finn-b-I9_AF_REIMB | Atrial fibrillation and flutter with reimbursement \|\| id:finn-b-I9_AF_REIMB | TRUE | reported | igd | 2 | TRUE |
| 19 | rs976568 | T | G | T | G | -0.1909 | -0.00447235 | 0.598 | 0.645804 | FALSE | FALSE | FALSE | ebi-a-GCST011364 | 4 | 111550721 | 0.01121297 | 17505 | 0.69 | Myocardial infarction \|\| id:ebi-a-GCST011364 | Myocardial infarction | Myocardial infarction \|\| \|\| | TRUE | igd | 0.0194 | 111550721 | NA | 6.08E-23 | 4 | finn-b-I9_AF_REIMB | Atrial fibrillation and flutter with reimbursement \|\| id:finn-b-I9_AF_REIMB | TRUE | reported | igd | 2 | TRUE |
| 20 | rs9825233 | T | C | T | C | 0.1185 | -0.000679172 | 0.5872 | 0.589663 | FALSE | FALSE | FALSE | ebi-a-GCST011364 | 3 | 12843368 | 0.010830918 | 17505 | 0.95 | Myocardial infarction \|\| id:ebi-a-GCST011364 | Myocardial infarction | Myocardial infarction \|\| \|\| | TRUE | igd | 0.0194 | 12843368 | NA | 1.02E-09 | 3 | finn-b-I9_AF_REIMB | Atrial fibrillation and flutter with reimbursement \|\| id:finn-b-I9_AF_REIMB | TRUE | reported | igd | 2 | TRUE |

Table 5

|  | **id.exposure** | **id.outcome** | **outcome** | **exposure** | **method** | **nsnp** | **b** | **se** | **pval** |
| --- | --- | --- | --- | --- | --- | --- | --- | --- | --- |
| 1 | ebi-a-GCST011364 | finn-b-I9_AF_REIMB | Atrial fibrillation and flutter with reimbursement \|\| id:finn-b-I9_AF_REIMB | Myocardial infarction \|\| id:ebi-a-GCST011364 | MR Egger | 30 | 0.39802972 | 0.1314332 | 0.00523588 |
| 2 | ebi-a-GCST011364 | finn-b-I9_AF_REIMB | Atrial fibrillation and flutter with reimbursement \|\| id:finn-b-I9_AF_REIMB | Myocardial infarction \|\| id:ebi-a-GCST011364 | Weighted median | 30 | 0.35170559 | 0.06768679 | 2.04E-07 |
| 3 | ebi-a-GCST011364 | finn-b-I9_AF_REIMB | Atrial fibrillation and flutter with reimbursement \|\| id:finn-b-I9_AF_REIMB | Myocardial infarction \|\| id:ebi-a-GCST011364 | Inverse variance weighted | 30 | 0.34921038 | 0.05651281 | 6.44E-10 |
| 4 | ebi-a-GCST011364 | finn-b-I9_AF_REIMB | Atrial fibrillation and flutter with reimbursement \|\| id:finn-b-I9_AF_REIMB | Myocardial infarction \|\| id:ebi-a-GCST011364 | Simple mode | 30 | 0.36461104 | 0.12243694 | 0.00580979 |
| 5 | ebi-a-GCST011364 | finn-b-I9_AF_REIMB | Atrial fibrillation and flutter with reimbursement \|\| id:finn-b-I9_AF_REIMB | Myocardial infarction \|\| id:ebi-a-GCST011364 | Weighted mode | 30 | 0.36461104 | 0.08876305 | 0.00029841 |

Table 6

|  | **id.exposure** | **id.outcome** | **outcome** | **exposure** | **method** | **nsnp** | **b** | **se** | **pval** |
| --- | --- | --- | --- | --- | --- | --- | --- | --- | --- |
| 1 | finn-b-I9_AF_REIMB | ebi-a-GCST011364 | Myocardial infarction \|\| id:ebi-a-GCST011364 | Atrial fibrillation and flutter with reimbursement \|\| id:finn-b-I9_AF_REIMB | MR Egger | 20 | -0.02519919 | 0.04470748 | 0.57995017 |
| 2 | finn-b-I9_AF_REIMB | ebi-a-GCST011364 | Myocardial infarction \|\| id:ebi-a-GCST011364 | Atrial fibrillation and flutter with reimbursement \|\| id:finn-b-I9_AF_REIMB | Weighted median | 20 | 0.016552343 | 0.02368338 | 0.48461371 |
| 3 | finn-b-I9_AF_REIMB | ebi-a-GCST011364 | Myocardial infarction \|\| id:ebi-a-GCST011364 | Atrial fibrillation and flutter with reimbursement \|\| id:finn-b-I9_AF_REIMB | Inverse variance weighted | 20 | 0.046510744 | 0.02186221 | 0.03338281 |
| 4 | finn-b-I9_AF_REIMB | ebi-a-GCST011364 | Myocardial infarction \|\| id:ebi-a-GCST011364 | Atrial fibrillation and flutter with reimbursement \|\| id:finn-b-I9_AF_REIMB | Simple mode | 20 | 0.012389914 | 0.0404171 | 0.762519 |
| 5 | finn-b-I9_AF_REIMB | ebi-a-GCST011364 | Myocardial infarction \|\| id:ebi-a-GCST011364 | Atrial fibrillation and flutter with reimbursement \|\| id:finn-b-I9_AF_REIMB | Weighted mode | 20 | 0.0152058 | 0.02872009 | 0.60262654 |
